# Authors’ Response to: Additional Measurement Approaches for Sleep Disturbances. Comment on “Transdiagnostic Self-management Web-Based App for Sleep Disturbance in Adolescents and Young Adults: Feasibility and Acceptability Study”

**DOI:** 10.2196/39198

**Published:** 2022-06-13

**Authors:** Colleen E Carney, Nicole E Carmona

**Affiliations:** 1 Toronto Metropolitan University Toronto, ON Canada

**Keywords:** youth, sleep, technology, mHealth, self-management, adolescents, young adults, mobile phone, smartphone, polysomnography

We thank Tsai and Liu [1] for their letter on our paper [2]. It is an interesting suggestion to include secondary measures of differing “objective” sleep constructs, such as electrical activity in a polysomnography (PSG), serum melatonin, glucose, or movement; however, prospective monitoring of habits and perception is the key measured construct in sleep health, as well as the diagnosis and treatment of insomnia. There was an elevated rate of insomnia symptoms in the sample, and it is important to remember that insomnia often does not have objective disturbance [3], as it is a disorder of self-reported complaint and experience. This is one of the reasons why PSG is not indicated for insomnia except to rule out other sleep disorders; other reasons include the fact that a single-night assessment is not useful, and in-lab testing can lead to dubious results because of conditioned arousal [4]. Data from the PSG is not used for an insomnia diagnosis nor is it used to determine if a treatment is effective; the patient does that.

To say that the study should be more quantitative negates that prospective sleep logging is both quantitative and the consensus tool for the field [5]. What matters in insomnia is the person’s perspective. Once we move into measurement of sleep via electrical activity, chemical activity, or movement, we are one step removed from the construct of interest—the person’s experience of sleeplessness. The idea to measure melatonin is somewhat curious, given that there is no reason to expect any melatonin issues to measure, or to detect an improvement in melatonin, unless there was a specific circadian rhythm disorder in the participant.

This seems a pertinent time to remind the authors of the letter [1] that these are nonclinical participants who want to improve their sleep with a self-management app. Sleep health is important for teenagers, and one would not expect anomalies in melatonin or the PSG in nonclinical users, nor would one be able to detect a change with such a restricted range (ie, these values are likely in the normal range). This is a self-management app for those with various sleep problems even in the subclinical range, and, therefore, the only aspect we could reasonably affect is the reason they would have used the app: to improve their perception of sleep. If a future researcher wanted to recruit a clinical sample, such as those with delayed sleep phase syndrome and complete assays (eg, dim light melatonin onset) or actigraphs, the app may be helpful in this regard, but given that it is self-management, and users can opt to ignore advice, it is unclear whether it would be effective in a clinical sample—again, this is for self-management purposes. Whereas some value an “objective” measure over self-report, it is important to consider the desired construct, the use of such data, and for whom the data applies: in this case, those who self-identify as wanting better sleep and are willing to track and change their sleep habits to improve their sleep experience. Our app, designed by experts and teenage users, was successful in this regard.

## Abbreviations

PSG: polysomnography

## References

[1] Tsai W-T, Liu T-L (2022). Additional measurement approaches for sleep disturbances. Comment on “A transdiagnostic self-management web-based app for sleep disturbance in adolescents and young adults: Feasibility and acceptability study”. JMIR Form Res.

[2] Carmona NE, Usyatynsky A, Kutana S, Corkum P, Henderson J, McShane K, Shapiro C, Sidani S, Stinson J, Carney CE (2021). A transdiagnostic self-management web-based app for sleep disturbance in adolescents and young adults: Feasibility and acceptability study. JMIR Form Res.

[3] Edinger JD, Fins AI, Glenn DM, Sullivan Jr RJ, Bastian LA, Marsh GR, Dailey D, Hope Tv, Young M, Shaw E, Vasilas D (2000). Insomnia and the eye of the beholder: Are there clinical markers of objective sleep disturbances among adults with and without insomnia complaints?. J Consult Clin Psychol.

[4] Littner Michael, Hirshkowitz Max, Kramer Milton, Kapen Sheldon, Anderson W McDowell, Bailey Dennis, Berry Richard B, Davila David, Johnson Stephen, Kushida Clete, Loube Daniel I, Wise Merrill, Woodson B Tucker, American Academy of Sleep Medicine, Standards of Practice Committe (2003). Practice parameters for using polysomnography to evaluate insomnia: an update. Sleep.

[5] Carney Colleen E, Buysse Daniel J, Ancoli-Israel Sonia, Edinger Jack D, Krystal Andrew D, Lichstein Kenneth L, Morin Charles M (2012). The consensus sleep diary: standardizing prospective sleep self-monitoring. Sleep.

